# Co-contamination of Aflatoxins with Ochratoxin A and Zearalenone in *Thuja orientalis* Semen

**DOI:** 10.5487/TR.2009.25.3.125

**Published:** 2009-09-01

**Authors:** So Yean Cho, Shin Jung Kang, Joohee Jung, Byeong Ok Jeong, Choon Sik Jeong

**Affiliations:** 13 Herbal Medicine Evaluation Team, Korea Food & Drug Administration, Seoul, 122-704 Korea; 23 College of Pharmacy, Duksung Women’s University, Seoul, 132-714 Korea

**Keywords:** Herbal medicines, Co-contamination, Aflatoxins, Ochratoxin A, Zearalenone, Thujae Semen

## Abstract

Korea is representative of a country that consumes herbal medicines; most of the herbal medicines circulating in South Korea have been imported from developing countries in Southeast Asia, such as China and Indonesia. Recently, domestic hygiene and safety are issues that have come to the forefront, because herbal medicines currently in circulation could possibly contain contaminants or residues. Furthermore, the appearance or discovery of harmful new species due to environmental and industrial developments is becoming a social problem. Therefore, it may be necessary to consider and investigate these matters on a continual basis. Recently, mycotoxin contaminations in such foods as cereals, nuts, and powdered red pepper have been reported. They have become a problematic issue; the possibility of contamination in herbal medicines has also been considered. Nevertheless, recognition of and research into mycotoxin contamination in herbal medicines has been scarce because herbal medicine is used in only a few nations. In this research, we identified contamination by aflatoxin which is known to be the most potent mutagenic, carcinogenic, and teratoge-nic mycotoxin in Thujae Semen, a herbal medicine. We also found co-contaminations involving other mycotoxins, including ochratoxin A and zeraleanone.

## INTRODUCTION

Thujae Semen is the dried ripe kernel of *Thuja orientalis* (Cupressaceae). It has been traditionally used in Oriental medicines to calm the nerves, check excessive perspiration, and relax the bowels. The ripe seed is collected in autumn and winter, dried in the sun, and removed from the testa. The Chinese Pharmacopoeia recommends that, to maintain its quality in herbal medicines, it should be stored in a cool, dry place and protected from heat and moths. In general, herbal medicines are stored or circulated in a dried state and at room temperature, according to traditional methods and, often, without adequate facilities that could otherwise prevent insect, animal, microorganism, and mycotoxin contaminations.

There are several factors involved in injurious changes to herbal medicine quality; temperature, humidity, insects, micro-organisms, residue, and contaminants are the most prevalent. Poor post-harvest management can lead to rapid deterioration in the nutritional quality of herbal medicines. Among them, microbial activity can cause undesirable effects in grains, including discoloration. It can also contribute to heating and losses in dry matter, by using carbohydrates as energy sources; degrade lipids and proteins, or alter their digestibility; produce volatile metabolites that give off odors; destroy the ability to germinate; and detrimentally affect baking and malting quality (Magan *et al*., 2004). Furthermore, mold growth also creates a potential human health risk, because of its production of toxic metabolites known as mycotoxins. Food and feed contamination with mycotoxins is a significant problem worldwide (Bennet and Klich, 2003); food contaminated by mycotoxins, when consumed by humans or animals, may cause mycotoxicosis and poisoning, resulting in death (Richard *et al*., 1993). Mycotoxin contaminations in herbal medicines may be unavoidable, as there are similarities between herbal medicines and corn, peanuts, tree nuts, and

**Table 1.** Limits of mycotoxins for herbal medicines in different countries

| Country | | Concentration (μg/kg) | | | |
|---|---|---|---|---|---|
| | | Aflatoxin $B_1$ | Total aflatoxins | Ochratoxin A | Zearalenone |
| Argentine | Herbs, herbal materials, herbal preparations used for herbal tea infusions | 5 | 20 | - | - |
| | Finished herbal products for internal or topical use | Absence per 1 gram | Absence per 1 gram | - | - |
| China | Herbal materials, extract and preparation | 5 | - | - | - |
| EU | Cpsicum spp., piper spp., Myristica fragrans, Zingiber officinale, Curcuma longa | 5 | 10 | - | - |
| | Devil's claw root, ginger, senna pods | 2 | 4 | - | - |
| Germany | Any materials used in manufacture of medicinal products (including medicinal herbal products) | 2 | 4 | - | - |
| Republic of Korea | Glycyrrhizae Radix Rhizoma, Cassiae Semen, Persicae Semen, Pinelliae Tuber, Thujae Semen, Arecae Semen, Zizyphi Semen, Polygalae Radix, Carthami Flos | 10 | - | - | - |

spices in terms of their respective circulating systems, storage conditions, and substrate characteristics, and the latter crops are known to be frequently contaminated by mycotoxins (Patel *et al*., 1996).

Mycotoxins are toxic secondary metabolites produced by certain mold species belonging to the genus types of *Aspergillus*, *Penicillium*, and *Fusarium*. *Aspergillus* and *Penicillium* are commonly known as storage molds; these mold species can grow post-harvest and during the drying and storage stages, especially when insufficient drying and unsuitable storage conditions favor their proliferation (Scott, 1984). While *Fusarium* species are destructive plant pathogens that produce mycotoxins pre- or immediately post-harvest, the *Aspergillus* and *Penicillium* species are more commonly found as contaminants of commodities and foods during the drying stage and subsequent storage (Sweeney and Dobson, 1998).

Some mycotoxins are found in fruits, vegetables, and botanical roots. These contaminants have a broad range of toxic effects, including acute toxicity, carcinogenicity, immunotoxicity, neurotoxicity, and reproducibility and developmental toxicity. Among the 400 known mycotoxins, aflatoxins are the most dangerous to human health, because of their highly toxic, carcinogenic, teratogenic, hepatotoxic, and mutagenic characteristics (Chu, 1997); they have been classified as group-1 carcinogens by the International Agency for Research on Cancer (IARC, 1999). They are produced by various strains of *Aspergillus* - mainly *A. flavus*, *A. parasiticus*, *A. tamari*, and *A. nominus* (Moss, 1998). Ochratoxin A is a secondary metabolite of certain *Aspergillus* and *Penicillium* species, such as *Aspergillus ochraceus* and *Penicillium verrucosum* (Meri *et al*., 2005). This toxin is responsible for nephrotoxic, immunosuppressive, and carcinogenic complications (Kuiper-Goodman and Scott, 1989), and it has been classified by the IARC as a possible human carcinogen (Group 2B) (IARC, 1993). Many authors have reported high levels of ochratoxin A in cereals, grain-based foodstuffs, and dried fruits (Gonzalez *et al*., 2006). Zearalenone is a non-steroid estrogen mycotoxin that is produced by numerous strains of *Fusarium graminearum* in wheat, barley, maize, and sorghum. This mycotoxin has an estrogenic effect on animals; its mutagenic, genotoxic, and carcinogenic effects remain elusive (Ouanes *et al*., 2005). It is classified by IARC as a group-3 carcinogen (IARC, 1999).

Contamination of herbal medicines with mycotoxins can cause harmful effects and serious problems, even in small amounts. There have been only a few reports regarding mycotoxin contamination of herbal medicines, because herbal medicines are used in only a few nations (Table 1). The use of herbal medicines as food or dietary supplements has increased, however, and so more research into and control of herbal medicines is required. Furthermore, because several mycotoxins frequently occur together, the detection of their combinations is an important problem. In this study, we found simultaneous contaminations of aflatoxins with ochratoxin A or zearalenone in Thujae Semen - through analyses with HPLC/UV or FLD- in aflatoxin-positive samples identified by ELISA and HPLC/FLD.

## MATERIALS AND METHODS

***Sample collection.*** Thujae Semen samples were randomly purchased during July and August 2006, from several shops in local Korean markets. Samples were collected from six different towns in the northern, central, and southern areas of Korea. All of the samples have been imported from China. Samples were stored in polyethylene bags at 4°C until analysis, and were returned to this temperature immediately after analysis.

***Chemicals and materials.*** All solvents were of HPLC grade, and purchased from Merck (Darmstadt, Germany); deionized water was used for the preparation of all aqueous solutions for HPLC. Ochratoxin A, aflatoxins, zearalenone, and ACS-grade acetic acid and nitric acid were purchased from Sigma-Aldrich (St. Louis, MO, USA). The Afla-Ochra-Zea immunoaffinity columns (AOZ HPLC$^{TM}$) and phosphate-buffered saline containing 0.01% Tween (pH 7.4) were obtained from Vicam (Watertown, MA, USA). All solvents employed were of glass-distilled or HPLC grade.

***Sample preparation and immunoaffinity column clean-up.*** A total of 5 g of powdered herbal medicine was added to a 100-ml volume of water:methanol mixture (30 : 70); it was then extracted through 30 min of sonication. The aqueous extract was filtered through folded filter paper. A 10 ml filtrated aliquot was added to 70 ml of water in a 150 ml conical flask. From there, 40 ml was loaded onto and passed through an immunoaffinity column at a flow rate of 3 ml/min (not exceeding 5 ml/min). After washing the column with two 10 ml volumes of water at a flow rate not exceeding 5 ml/min, the filtrate was dried by applying a slight vacuum for 5~10 s or by passing air through the immunoaffinity column, by means of a syringe, for 10 s. The mycotoxins were eluted by applying 0.5 ml of methanol to the column, which was allowed to pass through by gravity. The eluates were collected in a 5 ml volumetric flask. The second and third portions of 0.5 ml methanol were consecutively applied after 1 min. To remove any remaining water prior to quantitative elution, applied elution solvents were collected by pressing air through or applying vacuum to the column; the final eluates were diluted and shaken well with 5 ml of water, for HPLC analysis. If the solution was clear, it could be used immediately in analysis; otherwise, it was passed through a disposable filter unit (i.e., polytetrafluoroethylene filter with 0.45 mm pore size) that had been shown not to cause mycotoxin loss.

***Analysis of aflatoxins by HPLC.*** HPLC analysis was performed using an HPLC series 1100 from Agilent Technologies (Waldbronn, Germany), comprising a micro vacuum degasser, a binary capillary pump, a micro autosampler, and a column oven, as well as a fluorescence detector for aflatoxins. The chromatographic separation of aflatoxins was done with a CAPCELL-PAK ODS column (4.6 × 250 mm, 5 μm; SHISEIDO Co., Ltd., Tokyo, Japan). In the mobile phase, a acetonitrile : methanol : water mixture (2 : 3 : 6), including 350 μl dilute nitric acid and 0.12 g potassium bromide per liter, was applied. The flow rate was 1 ml/min, the column oven temperature was 30°C, and the injection column capacity was 20 μl. A Kobra Cell was used for post-column derivatization, while the fluorescence detector wavelength settings were 360 nm (excitation) and 445 nm (emission). Linearity was tested by injecting standard aflatoxin solutions at 10 concentration levels (ranges: $B_1$ and $G_1$, 0.01~100.5 ng/ml; $B_2$ and $G_2$, 0.02~25.0 ng/ml; three determinations were performed at each level). The significance of the linear regression and intercept (neither of which was significantly different from 0) was calculated via a *student's t*-test. Recovery experiments and calibration curves were obtained using the same concentration ranges. The recovery experiments were performed at three concentration levels (0.5, 1.0, and 2.0 μg/kg) by adding an appropriate amount of freshly prepared aflatoxin solution (100 μg/ml in acetonitrile) to the slurry. After spiking, the slurry was mixed and left overnight at room temperature (European Pharmacopoeia, 2007).

***Analysis of ochratoxin A by HPLC.*** An analytical determination of ochratoxin A was done after chromatographic separation, using a CAPCELLPAK ODS column (4.6 × 250 mm, 5 μm; SHISEIDO Co., Ltd.) at 30°C. The method included an isocratic mobile phase with 0.1% acetic acid:acetonitrile:methanol (5 : 4 : 1), at a flow rate of 1.0 ml/min and a run time of 20 min. The fluorescence detector wavelength settings were 333 nm (excitation) and 460 nm (emission). Linearity was tested by injecting ochratoxin A standard solutions at five concentration levels (range: 0.01~1.56 ng/ml; three determinations were performed at each level). The significance of the linear regression and intercept (neither of which was significantly different from 0) was calculated via a Student's t-test. Recovery experiments and calibration curves were obtained using the same concentration range. The recovery experiments were performed at three concentration levels (0.5, 1.0, and 2.0 μg/kg) by adding an appropriate amount of freshly prepared ochratoxin A solution (100 μg/ml in acetonitrile) to the slurry. After spiking, the slurry was mixed and left overnight at room temperature.

***Analysis of zearalenone by HPLC.*** Chromatography of zearalenone was done with CAPCELLPAK ODS (4.6 ×

250 mm, 5 μm; SHISEIDO Co., Ltd.) using an isocratic mobile phase with 0.1% acetic acid : acetonitrile : methanol (5 : 4 : 1) at 274 nm. The flow rate was 1.0 ml/min, the column oven temperature was 30°C, and the injection column capacity was 20 μl. The significance of the linear regression and intercept (neither of which was significantly different from 0) was calculated with a *student's t*-test. Recovery experiments and calibration curves were obtained using the same concentration range. The recovery experiments were performed at three concentration levels (0.5, 1.0, and 2.0 μg/g) by adding an appropriate amount of freshly prepared zearalenone solution (200 μg/ml in water) to the slurry. After spiking, the slurry was mixed and left overnight at room temperature.

## RESULTS

We have used in our study a one-step extraction and clean up process, determining simultaneous contaminations (featuring aflatoxins, ochratoxin A, and zearalenone) by HPLC, according to the Roswitha method (Roswitha and Klaus, 2004). It was, to some degree, very difficult to analyze simultaneous contaminations, owing to the different limits of detection (LODs) and mycotoxin contents in each sample. We therefore performed our one step process and then tried to determine each contaminant individually, by the best appropriate methods.

In the analysis of mycotoxins, there were no matrix interference peaks, and quite distinct from four aflatoxins, ochratoxin A, and zearalenone (Fig. 1). After cleanup with immunoaffinity columns, very "clean" chromatograms were obtained for herbal medicines that contained complex dyes or ingredients. No clearly visible peaks existed in the range of the retention times for mycotoxins.

For the analysis of aflatoxins, the LOD of aflatoxins $B_1$, $B_2$, $G_1$, and $G_2$ in Thujae Semen was 0.01 μg/kg. The limits of quantitation (LOQs) of aflatoxins $B_1$, $B_2$, $G_1$, and $G_2$ in Thujae Semen were 0.05, 0.02, 0.05, and

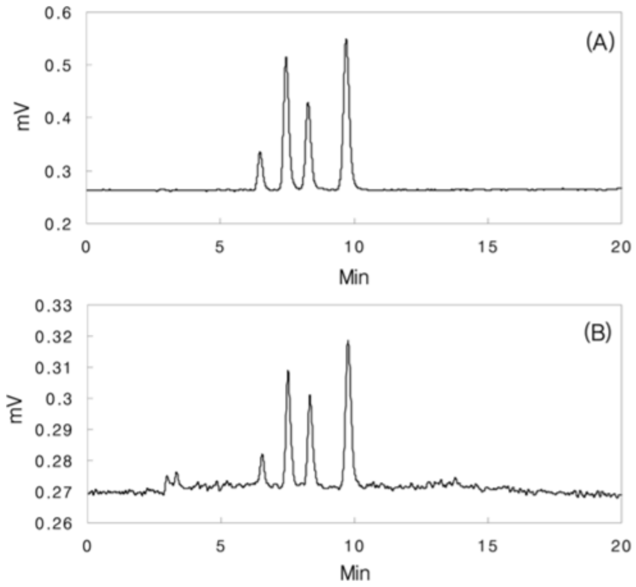

**Fig. 1.** Chromatogram of aflatoxins standard, $G_2$, $G_1$, $B_2$ and $B_1$ in order, and ochratoxin A contaminated Thujae Semen. Validation of mycotoxin determination by HPLC analysis. [a]Limit of detection (LOD), [b]Limit of quantification (LOQ).

0.05 μg/kg, respectively. The LOD and LOQ of ochratoxin A in Thujae Semen were 0.006 and 0.012 μg/kg, and 39.38 ng/kg and 157.5 ng/kg for zearalenone (Table 2), each respectively. All analysis methods showed good linearity, and a few matrix effects were observed within the same concentration range. Repeatability at both concentration levels was satisfactory, with the relative standard deviations (RSD) all being lower than 5.3%. Recovery experiments were performed within the spiking range of 0.5~2.0 μg/kg, with an average mean recovery of 79.5%. The significance of the linear regression and intercept (neither of which was significantly different from 0) was calculated via a *student's t*-test (Table 2).

Among all the samples, 41.7% were contaminated by aflatoxin $B_1$ concentrations above the regulatory limit of 10 μg/kg; samples had an average of 24.8 μg/kg of afla-

**Table 2.** Validation of mycotoxin determination by HPLC analysis

| Mycotoxins | LOD (μg/kg)[a] | LOQ (μg/kg)[b] | Regression | $R^2$ |
|---|---|---|---|---|
| Aflatoxin $B_1$ | 0.01 | 0.05 | Y = 1.8237X + 0.1484 | 0.9976 |
| Aflatoxin $B_2$ | 0.01 | 0.02 | Y = 4.8866X + 0.0665 | 0.9965 |
| Aflatoxin $G_1$ | 0.01 | 0.05 | Y = 1.7552X + 0.0532 | 0.9985 |
| Aflatoxin $G_2$ | 0.01 | 0.05 | Y = 2.2074X - 0.0176 | 0.9963 |
| Ochratoxin A | 0.006 | 0.012 | Y = 1.4082X - 3.4474 | 0.9962 |
| Zearalenone | 0.039 | 0.158 | Y = 48.327X + 0.3571 | 0.9998 |

[a]Limit of detection (LOD)
[b]Limit of quantification (LOQ)

**Table 3.** Contamination of mycotoxins in Thujae Semen

| Mycotoxins | Percentage of contamination | Range of contamination (μg/kg) | Mean (μg/kg) |
|---|---|---|---|
| Aflatoxin $B_1$ | 75.0 | 2.8 – 105.5 | 24.8 |
| Aflatoxin $B_2$ | 75.0 | 0.7 – 9.4 | 2.8 |
| Aflatoxin $G_1$ | 50.0 | 0.3 – 8.4 | 1.8 |
| Aflatoxin $G_2$ | 16.7 | 0.6 – 3.7 | 0.5 |
| Ochratoxin A | 22.2 | 49.8 – 54.9. | 52.1 |
| Zearalenone | 100 | 0.1-12.5 [a] | 8.4 [a] |

[a]mg/kg toxin $B_1$ and 27.9 μg/kg of total aflatoxins. Thujae Semen samples were contaminated by a wide range of concentrations, from 2.8 to 105.5 μg/kg of aflatoxin $B_1$ and 5.9~117.0 μg/kg of total aflatoxin (Table 3). Among the analyzed samples of Thujae Semen, 75% were found by HPLC analysis to contain aflatoxins; we then determined co-contamination by ochratoxin A or zearalenone in aflatoxin-positive samples. A total of 22.2% and 100% of the aflatoxin-positive samples were co-contaminated by ochratoxin A and zearalenone, respectively (Table 4). The range of ochratoxin A levels in Thujae Semen samples was between 50.0 and 54.2 μg/kg, with a mean content of 52.1 μg/kg. In Fig. 2, the chromatograms show the standard of ochratoxin A (A) and sample of Thujae Semen containing ochratoxin A. The chromatograms of zearalenone as a standard (A) and Thujae Semen sample (B) were shown in Fig. 3. The range of zearalenone levels in Thujae Semen samples was between 0.10 and 12.5 mg/kg, with a mean content of 8.4 mg/kg (Table 3).

## DISCUSSION

Mycotoxins can contaminate various agricultural products when their drying is delayed or their moisture levels exceed critical values *vis-à-vis* mold growth during storage (Saleemullah *et al.*, 2006). The cultivation, harvest, storage, circulation, and physico-chemical processes and properties of herbal medicines are very similar to those of agricultural products. Most herbal medicines are distributed to dry condition. In particular, herbal medicines are usually produced in countries with tropical or subtropical climates featuring high temperatures and high levels of humidity and rainfall. According to one study, mold contamination is often influenced by other factors, including insects, micro-organisms, and physical damages (D'Mello *et al.*, 1986). It is therefore possible for a commodity to be co-contaminated by two or more mycotoxins. Consequently, unknown factors or

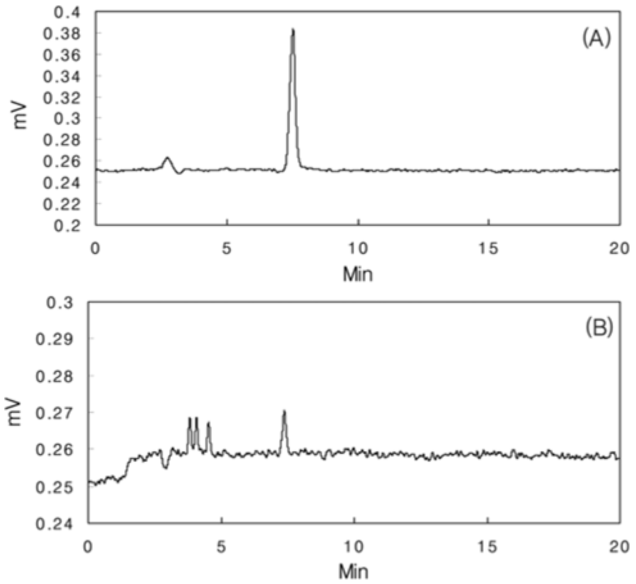

**Fig. 2.** Chromatogram of ochratoxin A standard and ochratoxin A contaminated Thujae Semen.

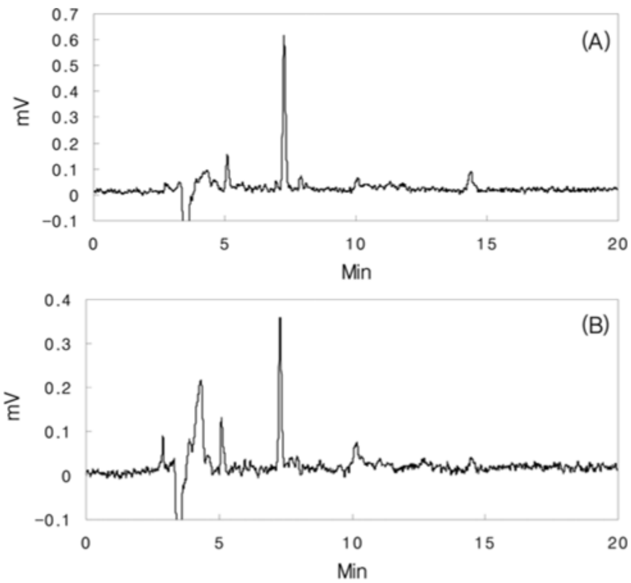

**Fig. 3.** Chromatograms of zearalenone standard and zearalenone contaminated Thujae Semen.

**Table 4.** Frequency of co-contamination of aflatoxins, ochratoxin A and zearalenone in Thujae Semen

| Frequency of mycotoxins co-contamination (%) | | | |
|---|---|---|---|
| Aflatoxins only | Aflatoxins and Ochratoxin A | Aflatoxins and Zearalenone | Aflatoxins, Ochratoxin A and Zearalenone |
| - | 22.2 | 100 | 22.2 |

synergistic effects of co-contamination may exist among these mycotoxins. In this study, the co-contamination of circulating Thujae Seman samples was investigated by HPLC/UV or FLD and ELISA. These results showed that the samples contaminated by aflatoxins were observed containing ochratoxin A (22.2%) or zearalenone (100%). The co-contamination data found from our study may be useful for the further study of synergistic effects on Thujae Semen.

It has been reported that 5~10% of agricultural products in the world are spoiled by mold contamination, to the extent that they cannot be consumed by humans or animals (Topal, 1993). Globalized trade in agricultural commodities has contributed significantly to discussions and awareness-raising *vis-à-vis* the potential hazards of mycotoxins. In addition, occurrences of mycotoxin contamination are likely to increase more and more by decisive factors such as increases in temperature and humidity from climate changes; the lack of quality control in herbal medication production; the use of traditional methods in product drying and storage; and the long periods of circulation and storage that often occur with herbal medicine products. The public health concerns related to both the acute and chronic effects of mycotoxins have prompted more than 100 countries to establish regulatory limits for some of the more prevalent mycotoxins. It is for this reason that the Korean Food & Drug Administration established in January 2008 standards and an experimental method for detecting aflatoxin $B_1$; its levels will be regulated, with a maximum level of 10 $\mu$g/kg being allowed in nine herbal medicines in which aflatoxins have been previously detected. Recently, Park and his colleagues reported that the contamination of aflatoxins in various herb medicines was detected (Park *et al.*, 2009). Although Thujae Semen was also included in his study, the contamination of aflatoxins only was reported. In this study, however, ochratoxin A and zearalenone as well as aflatoxins were simultaneously detected as a co-contaminator.

Mycotoxins are very harmful to human health and sanitation and detrimental to the quality of herbal medicines. It is important to control the quality of herbal medicines, because they are mainly consumed to improve overall health and treat or alleviate a wide range of diseases-often in individuals who are immunosuppressed, and who may therefore be unable to recover readily from mycotoxin poisoning. International research and regulations must be undertaken and established *vis-à-vis* decontamination methods against mycotoxins in herbal medicines, foodstuffs, and animal feed. Further studies are required, to evaluate the critical control points and develop those decontamination methods against micro-organisms and mycotoxins in the manufacture, circulation, and storage of herbal medicines.

## ACKNOWLEDGMENTS

This work is supported by research grants from the Korea Food & Drug Administration (2007), and by a Korea Research Foundation Grant funded by the Korean Government (MOEHRD) (KRF-2008-005-J00601).

## Abbreviations

HPLC/UV: high-performance liquid chromatography/ultra-violet
FLD: ferro liquid display
LOQs: limits of quantitation
LODs: limits of detection
RSD: relative standard deviations
